# Prevalence and risk factors of Influenza Avian Virus in backyard pigeons, ducks, and chickens in Toba Tek Singh District, Pakistan

**DOI:** 10.1371/journal.pone.0314186

**Published:** 2025-10-15

**Authors:** Iram Shakeel, Hamad Bin Rashid, Qurat ul Ain, Alijaan Inayat, Umer Shakeel, Adedayo Michael Awoniyi, Mamoona Chaudhry

**Affiliations:** 1 Department of Epidemiology and Public Health, University of Veterinary and Animal Sciences, Lahore, Pakistan; 2 Department of Community Health Sciences, The Aga Khan University Hospital Karachi, Karachi, Pakistan; 3 Institute of Collective Health, Federal University of Bahia, Salvador, Brazil; 4 Department of Veterinary Surgery, University of Veterinary and Animal Sciences, Lahore, Pakistan; 5 Department of Microbiology and Molecular Genetics MMG University of Okara, Okara, Pakistan,; 6 Faculty of Engineering and Applied Science, Riphah International University, Faisalabad, Pakistan,; 7 University of Edinburgh Medical School, College of Medicine and Veterinary Medicine, Scotland, United Kingdom,; 8 Zhejiang University-University of Edinburgh Institute, Zhejiang University School of Medicine, Haining, China; Sudan University of Science and Technology, College of Veterinary Medicine, SUDAN

## Abstract

Influenza Avian virus (IAV) is a zoonotic pathogen that can be transmitted from birds to humans. Multiple IAV pandemics have had a devastating impact on the poultry industry and backyard birds (including ducks, chickens, and pigeons) worldwide, notably in Europe, United States, Africa, and Asia. In Pakistan, numerous outbreaks of H7, H5, and H9 subtypes have been documented in both commercial and rural areas, resulting in significant financial losses. However, the epidemiological status of various IAV subtypes in backyard birds in rural areas remains largely unknown. This study aimed to evaluate the prevalence of IAV and associated risk factors among domesticated birds in the Toba Tek Singh District, Pakistan. A cross-sectional study was conducted between 2017 and 2019 using multistage cluster sampling approach. Pooled tracheal and cloacal swab samples were collected and tested for IAV. Positive pooled swab samples were subsequently evaluated at the individual level. RNA was extracted using theTrizol method, followed by multiplex RT‒PCR with specific primers and probes to detect the IAV M-gene and its subtypes. Statistical analysis was performed using a multivariable logistic regression model. Overall, the prevalence of IAV in backyard chickens, pigeons, and ducks was 13.4%, 7.7%, and 11.4%, respectively. The most commonly detected IAV subtypes included H7, H9, and HA/Untyped. No statistically significant difference (**p* *> 0.05) in IAV prevalence was observed across cities for any bird species. In the multivariable analysis, species type (particularly chickens and pigeons) was significantly associated with IAV prevalence, while fighting cocks showed a borderline association. Enhanced surveillance, improved biosecurity protocols, targeted educational initiatives, and the adoption of better farming practices are recommended to mitigate IAV transmission and safeguard both poultry production and public health in Pakistan.

## Introduction

Influenza Avian virus (IAV) is a zoonotic pathogen that is transmitted mainly among animals, especially birds, and occasionally to humans. There are four types of influenza viruses: A, B, C, and D, with only type A generally capable of causing global pandemics in humans [1]. IAV belongs to the family *Orthomyxoviridae*, genus *Alphainfluenzavirus, and* species *Alphainfluenzavirus influenzae* (formerly known as influenza A virus or FLUAV) [2]. The classification of influenza viruses is further divided into subtypes based on sixteen hemagglutinin (H1-H16) and nine neuraminidase (N1-N9) surface proteins found in avian species. Additionally, two new hemagglutinin subtypes, H17 and H18, have been found in bats in Africa, and H19 was more recently described in birds [2–4]. IAV exists in two main pathotypes: low-pathogenic avian influenza (LPAI) and high-pathogenic avian influenza (HPAI), which are associated with mild to severe disease and mortality in poultry. Subtypes H5 and H7 (HPAI) are linked with severe illness, high mortality rates, and devastating economic losses [5].

Over the past few decades, these viruses have caused repeated outbreaks globally, affecting both commercial and backyard poultry populations and, in some cases, spreading to humans, particularly in low- and middle-income countries (LMICs) across Asia and Africa [6,7]. The impact of infectious avian viruses is especially noticeable in countries with large poultry industries and extensive human-animal interactions. Notably, the first outbreak of HPAI (H7N3) in Pakistan was reported in 1994, resulting in the death of approximately 3 million birds and causing losses of over 6 million USD in the country’s agricultural sector [8]. Since then, several outbreaks involving H5, H7, and H9 subtypes have occurred, highlighting the virus’s persistence and vulnerabilities within the national poultry sector [9,10]. H9N2 subtype, in particular, has become endemic across Asia and is frequently isolated from both commercial and backyard poultry in Pakistan and its neighbouring countries [11–13].

Human infection with influenza virus can occur through direct contact with infected birds or animals during slaughter or processing, or indirectly via exposure to contaminated environments, such as drinking water or agricultural resources contaminated by secretions from infected birds [14]. The growing population in LMICs has driven a rapid expansion of the agricultural sector to meet increasing protein demands and create employment opportunities. In Pakistan, for instance, poultry is one of the fastest-growing segments of agriculture, with an estimated annual growth rate of approximately 10% and a production of nearly 20 billion eggs per year to help meet the country’s protein needs [15]. Consequently, backyard poultry farming has become a key source of livelihood in many rural communities, albeit with inadequate biosecurity measures, which pose potential risks of zoonotic spillover from animals to humans [16].

Wild and domestic birds play a significant role in the ecology of IAV. Ducks are established natural reservoirs, capable of maintaining and transmitting the virus without exhibiting symptoms. Surveillance during the 2016 H5N8 outbreak in Iran confirmed the presence of IAV in both commercial and backyard poultry, as well as in wild birds [17]. In Egypt, H5 was found to be endemic among ducks (2.4%), emphasizing the need for targeted prevention strategies [18]. While pigeons have been suggested as potential reservoirs, their role in transmission remains uncertain. These findings illustrate the complexity of IAV ecology and underscore the importance of incorporating multiple avian species into surveillance efforts [19].

Despite recurring outbreaks and potential associated risks, few studies have estimated the prevalence of IAV and its risk factors among domesticated birds (pigeons, ducks, and chickens) in Pakistan. Several risk factors have been linked to IAV transmission, including contact with infected domestic birds or contaminated environments, proximity to poultry farms, prior infection history, and exposure to wild birds [9,20]. In Pakistan, a large proportion of the population resides in rural areas, where backyard poultry farming is common in nearly every household, serving both subsistence and commercial purposes. This common practice presents a significant risk for viral persistence and zoonotic spillover to humans [9]. To address this gap, this study aimed to evaluate the prevalence of IAV and identify associated risk factors among domesticated birds in the Toba Tek Singh District, Pakistan. The findings should inform surveillance efforts, support outbreak prevention strategies, and guide the development of targeted interventions for vulnerable communities lacking satisfactory prevention measures.

## Materials and methods

### 2.1. Study area

The study was conducted in Toba Tek Singh District, situated in central Punjab, Pakistan. The district is bordered by Faisalabad to the east, Jhang to the west, and the Ravi River to the north. Geographically, it lies between 30°33’ and 31°2’ north latitude and 72°08’ and 72°48’ east longitude. This district consists of four tehsils (sub-district administrative units): Gojra, Kamalia, Toba Tek Singh, and Pir Mahal. The population of Toba Tek Singh District is approximately 2,191,495 [21], with the majority of livelihoods dependent on agriculture, livestock, and fishing. It is the second-largest poultry-farming hub in Pakistan [22], with an estimated poultry population of around 6.8 million birds. Most of these are unvaccinated and range in age from less than one month to five years. These birds are primarily raised in semi-cage systems for meat, egg production, or recreational purposes. The use of semi-cage and free-range poultry systems increases interactions with other animals and wild birds, thereby potentially heightening the risk of disease transmission.

### 2.2. Study design

We used multistage cluster sampling approach for the study. Toba Tek Singh District and its 4 tehsils: Toba Tek Singh, Gojra, Kamalia, and Pir Mahal were non-randomly selected as clusters, encompassing approximately 544 villages. Although the exact number of birds per household was unavailable, estimated poultry populations for each village were obtained from the Department of Livestock and Dairy Development, Punjab, Pakistan [23]. These estimates were instrumental in determining the sample size. Specifically, probability proportional to size with replacement was used to randomly select 30 villages as primary sampling units [24]. The sample size was calculated using C-Survey version 2.0, with a 95% confidence level and ±10% precision for the prevalence estimate. A design effect (DE) of 2 was applied to account for cluster sampling [9]. This approach was adopted due to the absence of a complete sampling frame for the population. Finally, eight households were systematically selected from each village for bird sampling using the spinning pencil method [25].

### 2.3. Sample collection

We conducted a cross-sectional study over two years, from 2017 to 2019. A total of 242 samples, comprising tracheal/oropharyngeal and cloacal swabs, were collected from healthy backyard birds. Five tracheal and three cloacal swabs were pooled, and positive pools were subsequently assessed at the individual level. The birds, including ducks, pigeons, and chickens, were sourced from villages in the Toba Tek Singh District. Samples were obtained from live birds using sterile swabs, with restraint provided by a trained specialist to ensure both animal welfare and sample integrity, in accordance with standard procedures. Swab samples were placed in brain heart infusion media supplemented with antibiotics and stored in labeled cryovials. Ice packs in an icebox were used to maintain the cold chain during transportation.

### 2.4. Questionnaire survey

The questionnaire included socio-demographic and poultry management-related variables, such as age, history of disease signs, proximity to commercial poultry farms, contact with wild birds, presence of fighting cocks, equines, dogs, and cats, mortality and recovery history, feeding practices, rearing conditions, family employment in commercial poultry farms, medication use, vaccination history, and water sources. Contact with wild birds and mammals, including cats and dogs, was considered due to evidence previously reported in the global literature [2]. The questionnaire was administered through face-to-face interviews with poultry owners in the selected villages. A structured, pre-designed questionnaire was used, and interviews were conducted only after obtaining informed consent from participants. To ensure clarity and data accuracy, the research team conducted regular visits to selected households, reviewing and finalizing responses on-site during data collection.

### 2.5. Virus propagation

All samples were aggregated into tracheal samples (chickens) and cloacal samples (pigeons and ducks), and pooled separately. Serum pathogen-free fertilized eggs were incubated at 37°C and 50–60% humidity and monitored for embryo development over 9–11 days using an egg candler. Subsequently, virus stock was inoculated into 9-day-old embryonated eggs and incubated at 37°C for 24–72 hours. After incubation, the allantoic fluid containing the virus was harvested from each egg and stored at −80°C after centrifugation at 12000 rpm for 10 minutes at 4°C [26].

### 2.6. RNA extraction method

We used Trizol method for RNA extraction from the allantoic fluid samples. A volume of 250 µL of fluid was mixed with 750 µL of Trizol agent by homogenization. The mixture was then mixed with 250 µL (100%) of chloroform, vortexed, and incubated at room temperature for 7 minutes. It was then centrifuged at 12000 rpm for 15 minutes at 4°C. Subsequently, 450 µL of the upper layer was carefully extracted and transferred to a new numbered vial. Next, 500 µL (100%) isopropanol was added, the mixture was incubated for 10 minutes at room temperature, and then centrifuged. The supernatant was discarded. After washing with 75% ethanol, the RNA precipitate was collected by centrifugation at 10,000 rpm for 8 minutes at 4°C, resuspended in 50 µL of RNase-free water, and stored following the methods previously described by Lee et al. [27].

### 2.7. RT‒PCR method

cDNA was synthesized from the extracted RNA using commercially available cDNA synthesis kit® **(Thermo Fisher Scientific, Lithuania)** following the manufacturer’s instructions. For general detection of IAV, primers and probes targeting the M-gene were used, as previously described by Lee et al. [27]. To differentiate between subtypes H5, H7 [28], and H9 [29], an additional RT-PCR assay was performed using subtype-specific primers targeting the HA gene segments. 25 µl RT-PCR reaction mixture contained 12.5 µl of PCR Master Mix, 2 µl of template, 1 µl of forward and reverse primers, and 8.5 µl of DEPC-treated water. Thermocycling conditions for M-gene amplification included cDNA synthesis at 45°C for 45 minutes, an initial denaturation at 95°C for 5 minutes, followed by 40 cycles of 95°C for 1 minute, 53°C for 1 minute annealing, and extension at 70°C for 1 minute. A final extension was performed at 72°C for 10 minutes, with a holding stage at 4°C. According to the protocol, negative and positive samples were used for the identification of differences.

### 2.8. Ethical considerations

Approval was obtained from both the Department Review Committee and the Ethical Review Committee of the University of Veterinary and Animal Science, Lahore. Informed consent was also obtained from all respondents before data collection. The confidentiality of all collected data was ensured through the implementation of standardized data protection protocols.

### 2.9. Statistical analysis

All data were recorded in an Excel spreadsheet and subsequently imported into Stata 16 for statistical analysis. Additionally, the geographical locations of selected households were recorded using a handheld global positioning system (GPS; Garmin, Olathe, KS, USA). Weighted point estimates of prevalence with 95% confidence intervals (CIs) in backyard birds within each city were calculated using the chi-squared test. Logistic regression with binary response variable (positive or negative IAV) was applied. Initially, 32 exploratory variables were screened using separate univariate logistic regression. Of these, 16 risk factors fulfilled the selection criterion (Wald test **p <* *0.25) and were included in the multivariable logistic regression analysis. Before model development, a collinearity test was conducted among the selected variables. A stepwise model-building approach was used to construct the final model, with variables retained or removed based on odds ratios and Wald test p-value ≤ 0.05. The final logistic model reported the odds ratios (ORs) and 95% CIs for all retained variables.

## Results

### 3.1. Prevalence of IAV in backyard birds

In this study, 13.1% prevalence of IAV was identified among backyard chickens. Toba Tek Singh exhibited the highest prevalence (14.8%), while Gojra had the lowest prevalence (11.5%). However, these differences were not statistically significant, suggesting a relatively uniform distribution of IAV among chickens across the four sampled cities (Table 1**).** Overall, the prevalence of H9 and H7 subtypes among backyard chickens was 10.3% and 2.8%, respectively.

**Table 1:** Positive tracheal samples of chicken for Influenza A virus and its subtypes (H7 and H9) detected by qRT-PCR in Toba Tek Singh District^*^.

Cities	Total Samples N	Positive AIV n (%)	95% CI for AIV positivity (%)	Subtype H7 n (%)	Subtype H9 n (%)
**Gojra**	26	3 (11.5)	2.5 - 29.1	1 (33.3)	2 (66.7)
**Toba Tek Singh**	27	4 (14.8)	5.9 - 32.2	0 (0)	4 (100)
**Kamalia**	26	3 (11.5)	2.6 - 30.2	1 (33.3)	2 (66.7)
**Pir Mahal**	28	4 (14.3)	5.7 - 31.5	1 (25)	3 (75)
**Total**	**107**	**14 (13.1)**	**7.9 - 21.4**	**3 (21.4)**	**11 (78.6)**

* There were no significant differences between the prevalence of Influenza A virus across cities among chickens (***p-*value=0.98**).

The prevalence of IAV in backyard ducks was 11.4%. Gojra showed the highest prevalence (13.3%), while Kamalia had the lowest prevalence (9.01%), but these differences were not statistically significance (Table 2**).** The overall prevalence of H7 and HA/Untyped subtypes among backyard ducks was 4.54% and 6.8%, respectively. Samples that tested positive for IAV but negative for H5, H9, and H7 subtypes were classified as IAV HA/Untyped.

**Table 2:** Positive cloacal samples of ducks for Influenza A virus and its subtypes (H7 and HA/Untyped) detected by qRT-PCR in Toba Tek Singh District^*^.

Cities	Total Sample N	Positive AIV n (%)	95% CI for AIV Positivity (%)	Subtype H7 n (%)	HA/Untyped n (%)
**Gojra**	15	2 (13.3)	1.7 - 40.5	1 (50)	1 (50)
**Toba Tek Singh**	8	1 (12.5)	0.3 - 52.7	0 (0)	1 (100)
**Kamalia**	11	1 (9.1)	0.2 - 41.3	1 (100)	0 (0)
**Pir Mahal**	10	1 (10)	0.3 - 44.5	0 (0)	1 (100)
**Total**	**44**	**5 (11.4)**	**3.8 - 24.6**	**2 (40)**	**3 (60)**

* There were no significant differences between the prevalence of Influenza A virus across cities among ducks (***p-*value=0.99**).

The prevalence of IAV in backyard pigeons was 7.7%. Toba Tek Singh reported the highest prevalence (9.5%), while Gojra reported the lowest prevalence (4.5%), although these differences were not statistically significant (Table 3). The overall prevalence of H7 and HA/Untyped subtypes among backyard pigeons was 6.6% and 1.1%, respectively.

**Table 3:** Positive cloacal samples of pigeons for Influenza A virus and its subtypes (H7 and HA/Untyped) detected by qRT-PCR in Toba Tek Singh District^*^.

Cities	Total Sample N	Positive AIV n (%)	95% CI for AIV Positivity (%)	Subtype H7 n (%)	HA/Untyped n (%)
**Gojra**	22	1 (4.5)	0.1 - 22.8	1 (100)	0 (0)
**Toba Tek Singh**	21	2 (9.5)	1.7-29.2	2 (100)	0 (0)
**Kamalia**	26	2(7.7)	1.4 - 25.1	1 (50)	1 (50)
**Pir Mahal**	22	2(9.1)	1.6 - 28.3	2 (100)	0 (0)
**Total**	**91**	**7 (7.7)**	**3.8 - 14.7**	**6 (86)**	**1 (14)**

* There were no significant differences between the prevalence of Influenza A virus across cities among ducks (***p-*value=0.97**).

**Fig 1:**
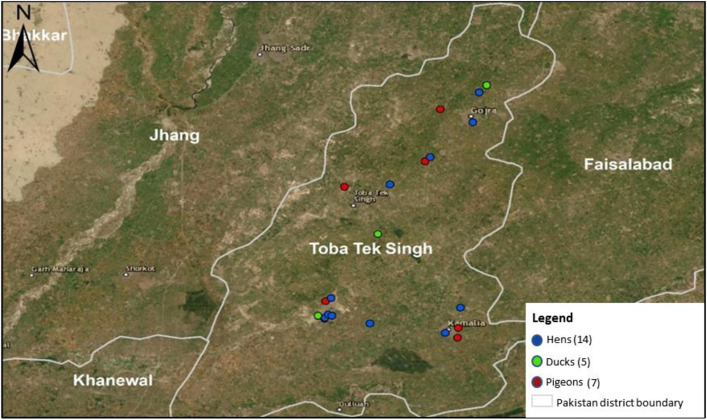
Map showing the location of positive IAV samples in ducks, pigeons, and chickens in Toba Tek Singh District. The map was created using **QGIS** 3.28 (Open-Source Geospatial Foundation). Cartographic sources: Territorial limits (**GADM**, 2022); Image (Google Satellite/Landsat/Copernicus, 2021). Geodetic Reference System: Kalianpur 1962.

**Fig 2:**
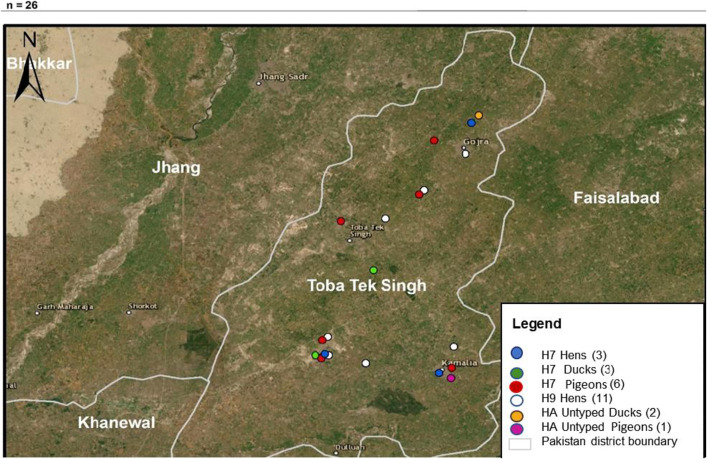
Map showing the location of positive H7, H9, and HA-Untyped IAV subtypes in ducks, pigeons, and chickens in Toba Tek Singh District. The map was created using **QGIS** 3.28 (Open-Source Geospatial Foundation). Cartographic sources: Territorial limits (**GADM**, 2022); Image (Google Satellite/Landsat/Copernicus, 2021). Geodetic Reference System: Kalianpur 1962.

Figs 1 and 2 show positive samples of IAV and its subtypes (H7, H9, and HA untyped) from ducks, pigeons, and hens in Toba Tek Singh District.

### 3.2. Risk factors associated with the prevalence of IAV

Initially, 46 variables were considered for univariate logistic regression. Based on their association with the study outcome (IAV-positive or IAV-negative birds), 32 variables (risk factors) were retained for the univariable analysis. Of these, 16 variables fulfilled the selection criterion (Wald test **p* *< 0.25) and were subsequently included in the multivariable logistic regression model **(see** S1 Table in the supporting information-named S1 Table).

According to the final model, two potential risk factors were significantly associated with the prevalence of IAV and its subtypes in backyard birds (p ≤ 0.05). Among the variables, species (pigeon OR 9.2, 95% CI: 1.92–44.2; p = 0.006) and (chicken OR 16.2, 95% CI: 2.89–91.5; p = 0.006) were strongly associated with the IAV prevalence among study communities. Additionally, the presence of fighting cocks showed a moderate association (OR 2.25, 95% CI: 0.99–5.09; p = 0.051) with IAV prevalence (Table 4**).**

**Table 4:** Results of multivariable analyses of risk factors for IAV prevalence in backyard birds in Toba Tek Singh District.

Risk Factors	Response Level	Positive Samples	Odds Ratio (OR)	CI (95%)	*p-*value
**Birds**	242	104			**0.006**
**Duck**	44	9	Ref		
**Pigeon**	91	39	9.21	1.92-44.2	
**Chicken**	107	56	16.2	2.89-91.5	
**Fighting Cock**	242	104			**0.051** ^*^
**No**	65	21	Ref		
**Yes**	177	83	2.25	0.99-5.09	

* There was an observed trend towards significant in the association between the IAV prevalence and fighting cock in the multivariate analyses **(*p-*value = 0.051**).

## Discussion

The increasing population of Pakistan has resulted in an increased demand for protein and intensified competition for limited resources. Consequently, residents across urban, peri-urban, and rural regions have increasingly invested in poultry farming, particularly in backyard and peri-domestic settings, often without adequate biosecurity protocols. This trend has contributed to a significant poultry mortality rate and a heightened risk of virus spillover from birds to humans.

The prevalence of IAV in chickens was 13.4%, with subtype-specific rates of 10.3% for H9 and 2.8% for H7. Among pigeons and ducks, the overall prevalence was 11.4% and 7.7%, with circulating H7 and untyped HA subtype detected in both species. Toba Tek Singh tehsil showed a higher prevalence compared to other communities, likely due to its dense population, diverse farming practices, and active poultry trade. Risk factor analysis indicated species type (P < 0.006) and the presence of fighting cocks (p = 0.051) as significant determinants of IAV infection. Chickens showed a strong association with IAV prevalence, with an odds ratio (OR) of 16.2 and a 95% CI of 2.89–91.5.

The observed prevalence of H9 (9.2%) and H7 (1.9%) in chickens aligns with previous reports from India, supporting the assertion that IAV is endemic in many Asian countries [30]. In contrast, our results are lower than those reported in Chile [31] and Vietnam [32], likely due to variations in sampling strategies, study populations, and sample sizes. Factors such as seasonal climate variations and the diverse poultry farming practices observed in Vietnam may have significantly influenced IAV transmission.

Our findings in pigeons and ducks align with previous results from China that reported similar prevalence rates for H7 and HA/untyped subtypes [33]. However, our results differ from studies conducted in Kosice [34] and Egypt [18], where higher prevalence rates were reported. Agro-environmental variables, such as rice cultivation landscapes and duck density, may play a crucial role in the ecology of IAV transmission in Southeast Asia, indicating that different landscape conditions may influence IAV transmission [35]. Additionally, cropping land coverage has also been suggested as an ecological factor facilitating the persistence and circulation of multiple HPAI viruses among poultry in Taiwan [36]. The absence of H5 in this study may be attributed to seasonal variation, migratory patterns, and ecological factors, influenced by geographic constraints. Pakistan’s arid climate and the prevalence of small, dispersed backyard flocks likely limit viral amplification compared to intensive poultry production systems. Furthermore, the temporal limitation of our sampling, restricted to a specific period rather than being conducted year-round, may have contributed to the absence of H5 detection [37].

The detection of hemagglutinin (HA)/untyped IAV subtypes in backyard poultry is a considerable concern, as these viruses may contribute to the emergence of novel or reassorted viral lineages that escape detection by current diagnostic tests. Their presence suggests an ongoing antigenic drift or shift process, involving mutations or genetic reassortment that lead to the development of virus variants with altered biological or immunological properties. These changes may enable the virus to evade immune responses, complicate control strategies, and increase the risk of zoonotic transmission. Systematic monitoring is essential for the early identification and prevention of disease outbreaks. Effective tracking of subtype evolution requires genomic surveillance, full-genome sequencing, and phylogenetic analysis, as well as the development of advanced diagnostic assays and novel vaccines. Considering the dynamic nature of IAV evolution, particularly in regions with high poultry density and frequent interactions between domestic and wild birds, sustained and comprehensive surveillance is necessary for mitigating public health risks.

Ducks are well-established reservoirs in the epidemiology of IAV, particularly playing a key role in the maintenance, evolution, and transmission of both LPAIV and HPAIV strains. Their migratory behavior and capacity for asymptomatic virus shedding make ducks potent vectors for spreading IAV from waterfowl to poultry, and potentially humans. This contributes to the global dissemination, prevalence, and genetic diversity of IAV strains [38]. The detection of H7 and untyped HA subtypes in ducks in this study underscores their role as important reservoirs of IAV. Their frequent interactions with wild waterfowl and access to shared, potentially contaminated water bodies could enhance the risks of virus transmission and raise serious zoonotic concerns [39,40]. Therefore, the implementation of robust biosecurity measures and active surveillance systems is essential to mitigate the spread of IAV and protect both animal and public health. Among the tehsils of Toba Tek Singh District, Toba Tek Singh exhibited a higher prevalence of IAV compared to other cities. This difference may be attributed to the denser population and variations in poultry farming practices, such as free-range farming, conventional farming, and semi-cage or cage-free farming, as well as the community’s prominent free trade and transportation networks in this region. The results from this study may help explain the previously observed high prevalence of IAV among poultry workers in Toba Tek Singh [41].

Over the past two decades, backyard poultry has significantly contributed to the income of many rural families in Pakistan, despite limited biosecurity conditions. A lack of awareness about IAV and its prevention strategies has enhanced farmers’ exposure to the virus. In this study, we identified the presence of fighting cocks as a significant risk factor (*p = 0.05*). Similarly, research in Thailand has shown an association between fighting cocks and IAV transmission. The regular movements of fighting cock owners, both nationally and internationally, may contribute to a heightened risk of disease transmission. To effectively manage IAV transmission, intervention programs should encompass awareness/education campaigns, movement restrictions, vaccination, disinfection of equipment, and the establishment of well-structured biosecurity systems in both rural and peri-urban areas. These interventions require consistent support from policymakers [42]. Moreover, monitoring the purchase, trade, congregation, and movement of fighting cocks is essential to limit the spread of IAV among backyard poultry at local and regional levels [43].

Species type also emerged as a significant risk factor (P < 0.006), as impoverished biosecurity practices in backyard flocks increase the likelihood of contact with wild waterfowl through shared feeding and water sources. Additionally, backyard birds may scavenge near crops and reservoirs alongside ducks, pigeons, and migratory species, increasing the probability of virus spillover to humans [44]. Chickens were strongly associated with IAV prevalence (OR 16.2, 95% CI: 2.89–91.5; *p < 0.006*), suggesting their role as a potential bridge for zoonotic transmission. Chicken is central to large-scale farming, serves as a primary protein source, and is heavily involved in the global poultry trade, a factor that increases the risk of IAV transmission [45]. Consequently, targeted monitoring and surveillance are essential to mitigate the risks of zoonotic spillover. The composition of avian species has a significant impact on infection outcomes [46,47]. Ducks, for example, exhibit positive selection for genes that suppress immune responses, leading to increased tolerance of both LPAI and HPAI. Conversely, chickens typically mount immune responses to LPAI, such as H9N2, but these responses offer minimal protective benefits against HPAI [48].

Although neither the univariate nor multivariable analyses in this study identified the presence of cats or dogs as predisposing risk factors, previous studies have suggested that animals such as cats, pigs, and dogs near poultry farms may contribute to IAV transmission [49,50]. Considering these species in future surveillance may help prevent secondary spread, especially following an initial outbreak, and could reduce the risk of widespread IAV pandemics and their associated economic impacts. This study has several strengths and limitations. Limitations include its cross-sectional design, relatively small sample size, and the inability to sequence untyped HA subtypes due to resource constraints, which may limit the generalizability and detailed characterization of circulating IAV strains. The strong association found in chickens (OR 16.2) should also be interpreted with caution due to wide confidence intervals, which may reflect sample size limitations. Nonetheless, the results provide valuable insights into the role of domestic birds in the transmission dynamics of IAV between avian and human populations. These findings should inform future prevention strategies and policymaking. Importantly, the use of RT-qPCR enhanced diagnostic specificity compared to serological testing. We recommend increased surveillance and molecular characterization of untyped HA subtypes in backyard poultry, particularly in rural and peri-urban areas of Pakistan, to mitigate potential zoonotic transmission.

## Conclusion

The high prevalence of IAV, particularly H7 and H9 subtypes, indicates its widespread distribution in Pakistan and highlights a significant risk of zoonotic transmission. Interactions between fighting cocks and other avian species at organized events likely facilitate the spread of the virus. Strengthening surveillance systems, enhancing biosecurity measures, providing targeted education for poultry farmers, and engaging policymakers in coordinated control strategies are essential components of effective disease management. These actions are crucial to protecting livelihoods, ensuring sustainable poultry farming, and enhancing outbreak preparedness in Pakistan and other similar LMICs with similar socio-environmental conditions. Such strategies could serve as models for LMICs with similar backyard poultry systems, including Bangladesh, India, Indonesia, Nigeria, Vietnam, and Kenya etc. helping them to prevent and respond to IAV outbreaks while ensuring sustainable poultry production.

## Supporting information

S1 Table Univariate logistic regression analysis. (DOCX)
