# Supplementary material for: Prevalence and risk factors of Influenza Avian Virus in backyard pigeons, ducks, and chickens in Toba Tek Singh District, Pakistan
Source: PLoS One. 2025 Oct 15;20(10):e0314186. doi: 10.1371/journal.pone.0314186 (PMC12527132; doi:10.1371/journal.pone.0314186)
Supplement: S1 Table — (DOCX) [file pone.0314186.s001.docx]

**Supplementary files**

**Prevalence and risk factors of Influenza Avian Virus in backyard pigeons, ducks, and chickens in Toba Tek Singh District, Pakistan**

Iram Shakeel, Hamad Bin Rashid, Quratul Ain, Alijaan Inayat, Umer Shakeel, Adedayo Michael Awoniyi, Mamoona Chaudhry

S1 Table. **Univariate logistic regression analysis**

**Results of univariate analyses of risk factors for AIV prevalence in backyard birds in District Toba Tek Singh ⃰**

| Factors | Response Level | Percentage  % | Positive Sample | Odd Ratio  (OR) | CI (95%) | *p-*value |
| --- | --- | --- | --- | --- | --- | --- |
| Birds  Duck  Pigeon  Chicken | 242  44  91  107 | 100  18.2  37.6  44.2 | 242  44  91  107 | Referent  9.21  16.2 | 1.92-44.2  2.89-91.5 | 0.01 |
| Type of breed  Mixed breed  Desi Breed | 242  126  116 | 100  52.1  47.9 | 104  56  48 | Referent  0.44 | 0.13-1.46 | 0.17 |
| Presence of Feline  No  Yes | 242  65  177 | 100  26.9  73.1 | 104  49  55 | Referent  2.78 | 0.79-9.74 | 0.10 |
| Distance from commercial poultry  Farm 1-3Km  Do not know  Less than 1Km  4-5Km | 242  68  41  64  69 | 100  28.1  16.9  26.4  28.5 | 104  26  22  30  26 | Referent  1.67  0.35  1.67 | 0.75-3.70  0.07-1.72  0.50-4.74 | 0.19 |
| Previous history of visiting any farm of your birds  No  Yes | 242  145  97 | 100  59.9  40.1 | 104  58  46 | Referent  2.72 | 0.90-8.14 | 0.07 |
| Respiratory illness  No  Yes | 242  156  86 | 100  64.5  35.5 | 104  64  40 | Referent  2.36 | 0.77-7.23 | 0.12 |
| Suddenly Death  No  Yes | 242  184  58 | 100  76.0  24.0 | 104  78  26 | Referent  1.91 | 0.71-5.11 | 0.18 |
| Purchase of younger birds  Govt  Hawker  Hatched at Home  Other  Commercial | 242  15  80  40  52  55 | 100  6.2  33.1  16.5  21.5  22.7 | 104  5  30  18  24  27 | Referent  0.84  0.32  1.58  1.99 | 0.25-2.85  0.04-2-15  0.49-5.09  0.73-5.38 | 0.016 |
| Wild Birds Contact  No  Yes | 242  95  147 | 100  39.9  60.7 | 104  44  60 | Referent  2.35 | 0.59-9.27 | 0.21 |
| Any family members or vehicles from commercial farm  No  Yes | 242  138  104 | 100  57.0  43.0 | 104  58  46 | Referent  0.44 | 0.12-1.68 | 0.20 |
| Disposed of your dead birds  Burn and bury  Open left | 242  58  184 | 100  24  76 | 104  24  80 | Referent  2.69 | 0.52-13.8 | 0.22 |
| Any pond canal stream near you  No  Yes | 242  100  142 | 100  41.3  58.7 | 104  46  58 | Referent  0.41 | 0.13-1.26 | 0.11 |
| Your birds have suddenly decreased production related to molting  No  Yes | 242  182  60 | 100  75.2  24.8 | 104  84  20 | Referent  0.58 | 0.31-1.07 | 0.08 |
| Buys adults birds  No  Yes | 242  177  65 | 100  73.1  26.9 | 104  73  31 | Referent  0.29 | 0.06-1.33 | 0.10 |
| History of Medication  No  Yes | 242  168  74 | 100  69.4  30.6 | 104  69  35 | Referent  2.64 | 0.82-8.42 | 0.097 |
| Fighting Cock  No  Yes | 242  65  177 | 100  26.9  73.1 | 104  21  83 | Referent  5.54 | 1.46-20.9 | 0.01 |

* The selection criterion (**Wald test *p*<0.25**) for univariate analyses for variables for multivariate analyses
